# Intra-crystalline protein diagenesis (IcPD) in *Patella vulgata*. Part I: Isolation and testing of the closed system

**DOI:** 10.1016/j.quageo.2012.03.016

**Published:** 2013-04

**Authors:** B. Demarchi, K. Rogers, D.A. Fa, C.J. Finlayson, N. Milner, K.E.H. Penkman

**Affiliations:** a BioArCh, Department of Chemistry, University of York, York YO10 5DD, UK; b Cranfield Forensic Institute, Cranfield University, Swindon SN6 8LA, UK; c The Gibraltar Museum, 18–20 Bomb House Lane, Gibraltar; d Department of Social Sciences, University of Toronto at Scarborough, Toronto, ON, Canada, M1C 1A4; e Department of Archaeology, University of York, King's Manor, York Y01 7EP, UK

**Keywords:** *Patella vulgata*, Intra-crystalline proteins, Mineralogy, Closed system, Leaching tests

## Abstract

This study successfully isolates a fraction of intra-crystalline proteins from shells of the marine gastropod *Patella vulgata* and assesses the suitability of these proteins for IcPD (Intra-crystalline Protein Diagenesis) geochronology. We discuss the mineralogical composition of this gastropod, investigated for the first time by X-ray diffraction mapping, and use the results to inform our sampling strategy. The potential of the calcitic rim and of a bulk sample (containing both apex and rim) of the shell to act as stable repositories for the intra-crystalline proteins during diagenesis is examined. The composition and the diagenetic behaviour of the intra-crystalline proteins isolated from different locations within the shell are compared, highlighting the necessity of targeting consistent sampling positions.

We induced artificial diagenesis of both intra-crystalline and whole-shell proteins by conducting high-temperature experiments in hydrous environment; this allowed us to quantify the loss of amino acids by leaching and therefore evaluate the open- or closed-system behaviour of the different fractions of proteins. The results obtained provide further confirmation that patterns of diagenesis vary according to the protein sequence, structure, and location within or outside the intra-crystalline fraction. As *Patella* is frequently found in the fossil record, both in archaeological and geological contexts, the application of IcPD geochronology to this biomineral opens up the possibility to obtain reliable age information from a range of sites in different areas of the world.

## Highlights

► Successful isolation of an intra-crystalline fraction of proteins from *Patella vulgata*. ► Investigation of the mineralogical composition of *P. vulgata* by X-ray diffraction. ► Optimisation of the sampling strategy for AAR geochronology on *P. vulgata*. ► Testing of the intra-crystalline proteins as a closed system during diagenesis. ► *P. vulgata* is shown as a suitable substrate for IcPD geochronology.

## 1 Introduction

Isolating a closed system of proteins within biominerals enables the study of the diagenesis undergone *in situ* by the original protein fraction (Curry et al., 1991). As protein breakdown within such a system should be solely dependent on time and temperature, the extent of degradation can be considered an accurate indicator of the thermal age of a sub-fossil sample. This has powerful implications for protein diagenesis/amino acid racemisation geochronology (AAR). Here we use the general term *racemisation* to indicate the post-mortem interconversion between two chiral forms of an amino acid; the extent of racemisation is quantified as the ratio between the concentrations of the D- and L-enantiomers (D/L value) or, for isoleucine, between the two diastereoisomers D-alloisoleucine and L-isoleucine (A/I value).

An indigenous intra-crystalline fraction of proteins, behaving as a closed system with regards to diagenesis, has been reported in eggshells (Brooks et al., 1990) and has been isolated by a strong oxidation treatment in a range of other calcareous biominerals (Penkman et al., 2008, 2011; Demarchi, 2009; Hendy et al., 2012; Crisp et al., 2013; Tomiak et al., 2013). Ostracodes have also been tested for their ability to retain an intra-crystalline fraction of proteins which can be isolated by strong oxidation; however, proteins in this biomineral are intimately associated with an abundant chitinaceous component, which results in a pool of proteins immune to chemical removal, hampering the effectiveness of the oxidation treatment (Bright and Kaufman, 2011).

The new methodology of intra-crystalline protein diagenesis (IcPD) geochronology has been recently applied to calcitic opercula of the gastropod *Bithynia*, resulting in a reliable chronostratigraphic framework for the Quaternary of the British (Penkman et al., 2011). It is believed that the success of this application to opercula depends upon both the isolation of the intra-crystalline proteins and the targeting of a calcitic biomineral, which is inherently less prone to mineral diagenesis and therefore offers a better protection for the intra-crystalline proteins (Penkman et al., 2010). As a consequence, we attempted the isolation of intra-crystalline proteins from other biominerals with calcitic mineralogy; for marine molluscs, we initially focused on the gastropod *Patella vulgata*.

*P. vulgata* was chosen to test the IcPD geochronological approach due to its wide geographical distribution, its abundance in archaeological contexts (as well as in relict beach deposits) and on its usefulness as a palaeoenvironmental indicator (e.g. Straus and Clark, 1986; Bailey and Craighead, 2003; Finlayson et al., 2006; Ferguson et al., 2011). Due to their ubiquity in the fossil record, these cone-shaped gastropods can offer the means for comparative study of archaeological and environmental phenomena across both the temporal and the spatial scales. Unsurprisingly, *Patella* shells have been a material of choice for AAR geochronology in coastal environments (Andrews et al., 1979; Davies, 1983; Bowen et al., 1985; Ortiz et al., 2009).

As opposed to the simple structure of opercula, shells of the genus *Patella* display wide microstructural diversity between species: up to six different layers can be present in a single shell, with different structures (e.g. prismatic, foliated, crossed and complex crossed-lamellar) and mineralogies (calcite and/or aragonite) (MacClintock, 1967). After the death of the organism, over time the thermodynamically less stable aragonite can undergo diagenesis to form calcite (Benton, 2001). This has an impact of the integrity of the intra-crystalline proteins as a closed system (Penkman et al., 2007, 2010) and therefore could be a key issue in the IcPD of *Patella* shells. As a consequence, we aimed to isolate a portion of the shell which is composed exclusively of primary calcite, i.e. calcite which has not undergone diagenetic recrystallization. Although the distribution of calcitic and aragonitic layers within *Patella* (and other molluscs of the order Patellogastropoda) is generally known (MacClintock, 1967; Fenger et al., 2007; Ortiz et al., 2009; Suzuki et al., 2010), in this study we complemented the available information by performing X-ray diffraction (XRD) mapping on both modern and archaeological shells, in order to obtain high-resolution spatial data which guided our sampling strategy for this taxon.

This paper provides the background to the successful application of IcPD geochronology to archaeological shells (e.g. Demarchi et al., 2011), reporting the systematic study of the behaviour of the intra-crystalline proteins within *Patella* by:

• Investigating the mineralogy by XRD in order to select calcitic portions of the shells, hypothesised to have greater potential for the preservation of the intra-crystalline proteins as a closed system;
• Isolating the proteins from both “bulk” shell powder and shell rim only (bleaching tests);
• Testing the robustness of the intra-crystalline fraction during artificial diagenesis (leaching tests at high temperature);
• Comparing the diagenetic patterns within the intra-crystalline fraction and within the whole-shell proteins.

## 2 Materials and methods

### 2.1 Materials

Five *P. vulgata* shells (SM1–5) were collected live at St Mary’s Lighthouse, Newcastle (UK) in 2001 (see also the study by Fenger et al., 2007, where *Patella* specimens from the same location were targeted for oxygen isotope analyses). One modern *P. vulgata* shell (C2) was collected dead from the shore at Robin Hood’s Bay, Yorkshire (UK), in 2007. Two archaeological specimens (NEaar laboratory I.D.: LOT 2035 and LOT 2045) from the Middle Palaeolithic layers of Vanguard Cave in Gibraltar were also included in this study. Specimen 2045 comes from a shell midden deposit in the Upper Area of Vanguard cave (see Stringer et al., 2008); specimen 2035 comes from material removed during the cleaning of the archaeological sections within the same cave. Although numeric age information for these two shells is not available, the deposits within Vanguard Cave have been estimated to be older than 41 ka BP, when the cave was infilled by sediments and sealed (Pettitt and Bailey, 2000; Stringer et al., 2000, 2008).

Two modern specimens (SM3 and C2) and the two archaeological shells (LOTs 2035 and 2045) were selected for XRD analyses. Cross-sections of 3–5 mm thickness were prepared by cutting with a diamond-blade saw along the major axis of each shell. Modern specimens only (SM1–5) were targeted for AAR analyses and prepared as described in Section 2.3.

### 2.2 XRD analysis

XRD analyses were performed on cross-sections of *Patella* specimens (XRD mapping) and on one powdered subsample from the shell rim only (powder XRD). For XRD mapping on the cross-sections data acquisition was performed by a mapping diffractometer (Cranfield University, Shrivenham, UK). Primary data was collected using a Bruker D8 Discover with GADDS (General Area Detector Diffraction System) and CuKα radiation. The experimental design used in this study allowed the mapping of different mineral phases along a chosen interrogation line, with steps of 50 μm and a 50 μm monocapillary. Diffraction patterns were obtained on two different areas of the shells (the apex and the rim) in order to detect the distribution of calcite and aragonite in the specimens.

XRD analyses were performed on the powdered shell rim sample (LOT 2035) following the methods of Batchelder and Cressey (1998) (Natural History Museum, London, UK).

### 2.3 Amino acid analysis

For AAR analysis, each *P. vulgata* shell (SM 1–5) was sonicated and rinsed five times in ultrapure water (18 mΩ), then air-dried and crushed with a quartz mortar and pestle. Two different batches of powdered shell were prepared:

• A “bulk” powder batch, where, from each of the five specimens, one fragment (less than 1 cm^2^) from the shell apex and one from the shell rim were taken (for a total of 10 fragments) and powdered together (subsampling positions are shown in Fig. 2);
• A “rim only” batch, where one fragment (less than 1 cm^2^) was taken from the rim of each of the five shells, as shown in Fig. 2 and detailed in Section 3.1.1, and the five fragments were then powdered together.

These powders were used for bleaching and leaching tests. Here we briefly describe the preparation protocols and the experimental set up used for both experiments, as well as the analytical method of detection of the chiral amino acids.

#### 2.3.1 Bleaching tests

NaOCl (sodium hypochlorite/“bleach”, 12% w/v) has been reported as an effective oxidising agent, able to remove the matrix proteins and isolate the intra-crystalline fraction in a range of biominerals (Penkman et al., 2008; Crisp et al., 2013; Tomiak et al., 2013). We tested the effectiveness of bleaching on *Patella* by exposing three particle sizes (fine: 50–100 μm; medium: 100–500 μm; coarse: 500–1000 μm) of powdered “bulk” shell to NaOCl for increasing times (0, 8, 18, 24, 48, 98, 120, 240 h). For the “rim only” batch, the medium-to-fine fraction only was selected and exposed to the oxidising agent for 48 h.

For each fraction, an excess of bleach (50 μL per mg of powdered shell) was added to accurately weighed subsamples at room temperature. Three replicates for each time point were prepared. The tubes containing the powders and the bleach were shaken every 24 h, in order to ensure the complete penetration of the oxidising agent. The bleach was then taken off and the powders rinsed five times in ultrapure water, once in HPLC-grade methanol (to destroy any residual oxidant by reaction with the methanol). Each rinse was performed by adding the liquid to the powders and then centrifuging for 3 min, to minimise the removal of powder while the liquid was pipetted off. Finally, the samples were air-dried overnight and split into two subsamples (FAAp and THAAp), for the analysis of the free and total hydrolysable amino acid fractions; free amino acids are released by natural hydrolysis, and total hydrolysable amino acids are released from the peptide bonds by preparative acid hydrolysis.

#### 2.3.2 Leaching tests

Leaching tests were performed by inducing artificial diagenesis to proteins within *Patella* by exposing them to high temperatures. The main leaching experiment was carried out at 140 °C (see Table 1) for the unbleached and bleached “bulk” powder batch and for the bleached “rim only” powder batch; in both cases, the medium-to-fine powder fraction only was selected, because they yielded more consistent results than the coarse powders when exposed to the bleaching agent (see Section 3.2.2). Additional data was obtained for the bleached “bulk” batch only, on samples heated isothermally at 110 °C and 80 °C (see Supplementary Information). Unbleached powders were not tested at 110 °C and 80 °C because it was assumed that the greatest leaching of amino acids in the water would occur at the highest temperature.

**Fig. 1:**
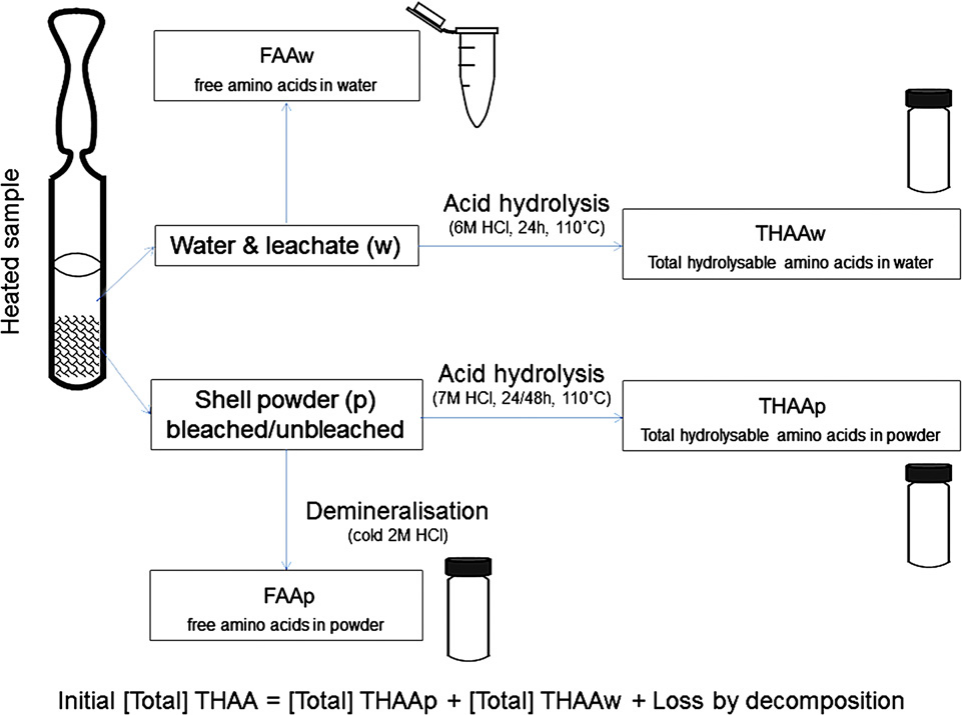
Schematic representation of the sample preparation protocol used for high-temperature experiments: unbleached and bleached *Patella* powders are heated at 140 °C in sealed sterile glass ampoules; after heating, both the water (w) and the powder (p) fractions are split in two aliquots (water: FAAw and THAAw; powder: FAAp and THAAp) and prepared for amino acid analysis. FAAw samples are dried in centrifugal evaporator; THAAw samples are hydrolysed in 6 M HCl at 110 °C for 24 h and dried in centrifugal evaporator; FAAp samples are demineralised in cold 2 M HCl and dried in centrifugal evaporator; THAAp samples are hydrolysed in 7 M HCl at 110° for 24 h (or 48 h, as discussed in Section 3.2.1) and dried in centrifugal evaporator. After these preparative steps, all samples are rehydrated using an aqueous solution containing a known concentration of the internal standard, the non-protein amino acid L-homo-arginine.

All samples were prepared following the protocol of Penkman et al. (2008), shown in the scheme in Fig. 1. In brief, ∼20 mg aliquots of dried powder were weighed out accurately and placed in individual sterile glass ampoules. 300 μL of ultrapure water (18 mΩ) were added to each ampoule, which was then sealed and placed in the oven for various times and at different temperatures (Table 1). Three laboratory replicates were prepared for each time point. After heating, 100 μL of the supernatant water (w) was analysed for the free amino acids leached into the water (FAAw) and 100 μL analysed for the THAA fraction (THAAw). Similarly, the dried powders (p) were divided into two subsamples (FAAp and THAAp), accurately weighed and placed in individual sterile glass hydrolysis vials (Fig. 1).

#### 2.3.3 Chiral amino acid analysis

For all samples obtained from the bleaching and leaching experiments, both FAA and THAA fractions were measured routinely (see Fig. 1). FAAp samples were demineralised in the minimum volume possible of cold 2 M HCl; THAAp were hydrolysed in 7 M HCl (20 μL/mg), usually for 24 h at 110 °C (however, the effectiveness of this hydrolysis step was tested by hydrolysing some of the bleached and unbleached *Patella* samples for 48 h, see Section 3.2.1); THAAw samples were hydrolysed in 6 M HCl (20 μL/mg equivalent, see Penkman et al., 2008) for 24 h at 110 °C; all samples were then dried in a centrifugal evaporator overnight. FAAw samples were dried in centrifugal evaporator directly after heating.

All samples were then rehydrated (usually 10–20 μL/mg of original sample) with the rehydration fluid routinely used in the NEaar laboratory, a solution containing an internal standard of the non-protein amino acid L-homo-arginine. The chiral forms were separated and detected on an automated reverse phase high pressure liquid chromatography (RP-HPLC) system, following an adapted analytical method of Kaufman and Manley (1998). Procedural blanks for each experiment were routinely prepared and analysed. In this study we focused on the 9 amino acids for which we obtained optimal chromatographic resolution: Asx (aspartic acid/asparagine), Glx (glutamic acid/glutamine), Ser (serine), Gly (glycine), Ala (alanine), Val (valine), Phe (phenylalanine), Leu (leucine), Ile (isoleucine).

## 3 Results and discussion

### 3.1 XRD analyses

#### 3.1.1 Shell layer mineralogy

XRD mapping was performed to investigate the distribution of calcite and aragonite bands in *P. vulgata* shells, with the aim of establishing the most appropriate sampling procedure for this marine gastropod, i.e. the isolation of a layer where primary calcite only is present.

**Fig. 2:**
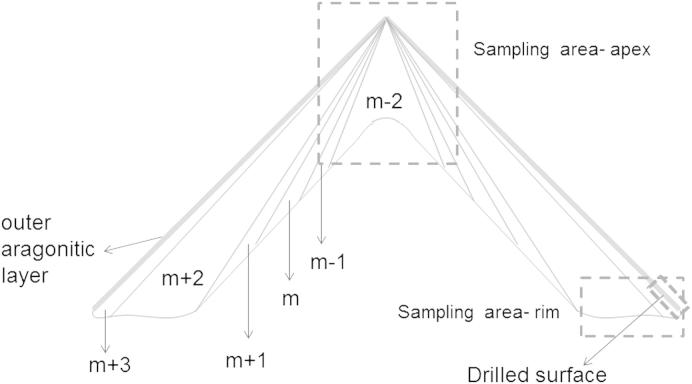
Schematic representation of the shell layers of *P. vulgata* (after MacClintock, 1967): *m* + 3, *m* + 2 and *m* − 2 are composed of calcite; *m* + 1, *m* and *m* − 1 of aragonite. The thin outer aragonitic layer detected by XRD analysis in this study is also shown (thick grey line). Dashed lines indicate the sampling areas chosen for this study (apex and rim) and the removal of the outer aragonitic layer.

XRD does not allow discrimination between the different microstructures of the layers, but is able to detect the presence of small amounts of the two polymorphs (>1 wt%). Shell layers are generally named according to their position relative to the myostracum (m), the material deposited adjacent to the mantle epithelium cells, where the area of the muscle insertion is found (Fig. 2). Layers which lie dorsally to this will be named *m* + 1, *m* + 2, …, *m* + n; while layers lying ventrally will be termed *m* − 1, *m* − 2 and so forth. MacClintock (1967) recognised five different microstructures across the shell layers in *P. vulgata*: radial crossed-foliated (*m* + 3, calcitic), concentric crossed-foliated (*m* + 2, calcitic), concentric crossed-lamellar (*m* + 1, aragonitic), radial crossed-lamellar (*m* − 1, aragonitic) and irregularly foliated to radial crossed-foliated (*m* − 2, calcitic). The position and the thickness of the aragonitic layers (*m*, *m* + 1 and *m* − 1) within *Patella* have been investigated in a number of studies, by both scanning electron microscopy (SEM) and visible light microscopy, typically used in combination with aragonite-specific staining solutions such as Feigl’s (MacClintock, 1967; Bailey and Craighead, 2003; Fenger et al., 2007; Ortiz et al., 2009; Ferguson et al., 2011) (Fig. 2).

**Fig. 3:**
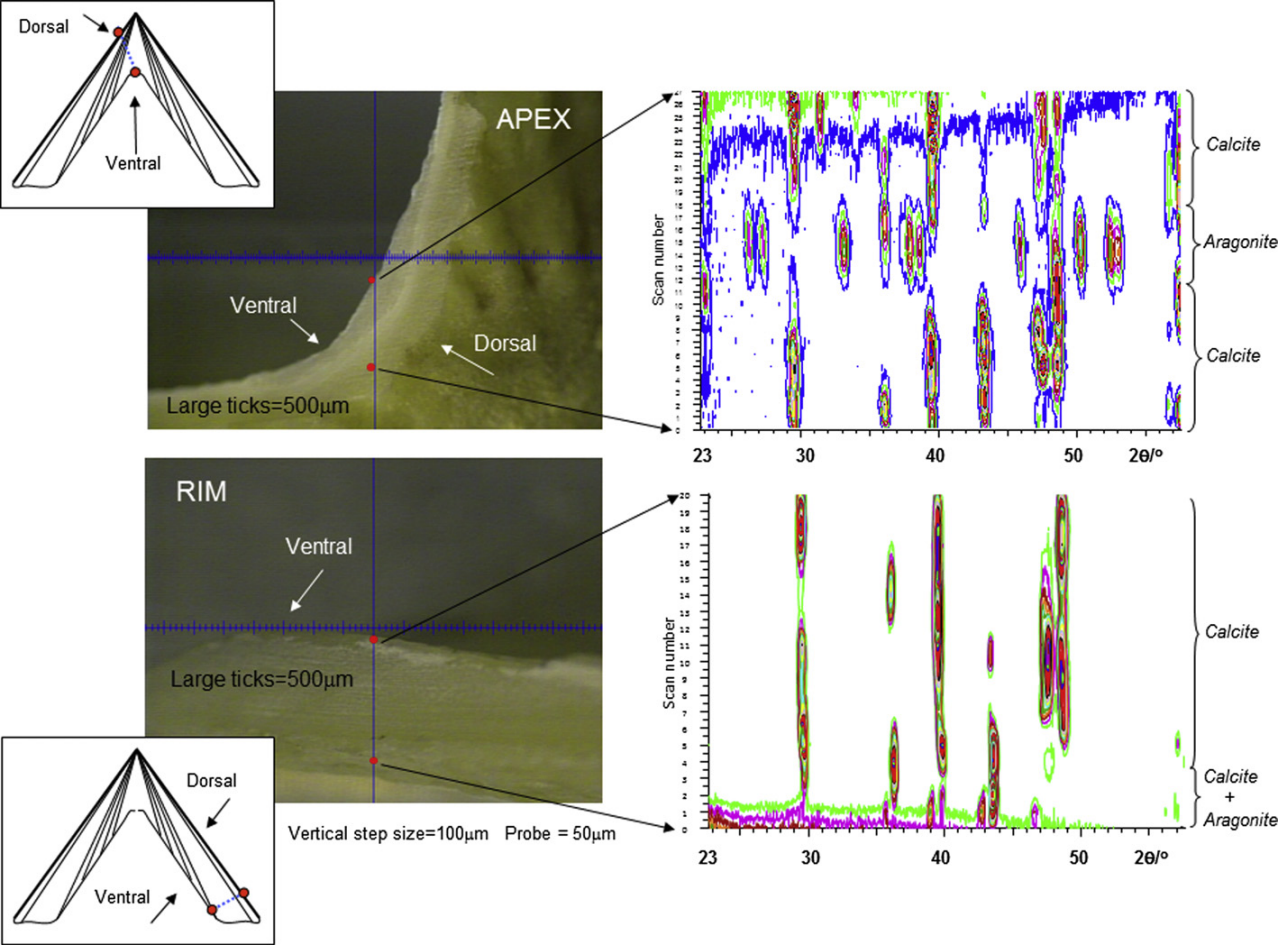
XRD diffraction patterns obtained on the archaeological *P. vulgata* LOT 2035 (provenance: Vanguard cave, Gibraltar), showing the apex (top) and rim (bottom). Note the presence of an internal band of aragonite within the apex.

The XRD mapping analyses performed in this study on modern and archaeological *P. vulgata* shells showed the presence of a thin (less than 10 μm) outer layer of aragonite in the modern apices and fossil rims. The outermost layer in *Patella* (*m* + 3) is composed of calcite (e.g. Fenger et al., 2007) therefore the presence of aragonite on the shell surface cannot be interpreted as layer *m* + 3. Although the aragonite could derive from contamination from the exoskeleton of other living organism attaching the shell surface, we did not find supporting evidence for this when the specimens were visually examined prior to analysis. Apart from the thin outer layer, only calcite was detected in the rim of all the samples analysed. Aragonite was found as an internal band within the apex of both archaeological shells, but not in the apex of modern shells (Fig. 3).

Our findings agree with those of MacClintock (1967), Fenger et al. (2007) and Ortiz et al. (2009) with regard to the distribution of the aragonitic and calcitic layers in *P. vulgata*. The calcitic layers detected in the shell rims of the four specimens should correspond to layers *m* + 2 and *m* + 3, concentric crossed-foliated and radial crossed-foliated, respectively; the presence of the two different microstructures is shown by the change in the preferred orientation between these layers which results in significant modifications to the relative intensities of the Bragg peaks (Fig. 3).

The interrogation line chosen for the apex of shell SM3 showed the presence of calcite only; specimen C2, although it presented an “obscured” region along the interrogation line for XRD analysis, also did not show any presence of aragonite in the apex area. This thick calcitic area can be interpreted as the innermost layer *m* − 2, present only in species *P. vulgata* among the genus *Patella*; in *Patella* the layers become thin towards the apex of the shell, with the exception of the innermost layer which has its extreme thickness in the apical region (MacClintock, 1967; Fenger et al., 2007). Similarly, Ortiz et al. (2009) highlighted the presence of a thick calcitic layer in the apex of a range of archaeological *Patella* shells stained with the Feigl’s solution. Conversely, the detection of aragonite within the apices of specimens LOT 2035 and LOT 2045 suggests that layers *m*, *m* − 1 and/or *m* + 1 were present in these archaeological shells (Fig. 3).

These results highlight the high intra-specific natural variability displayed by *P. vulgata*. Limpets can live up to 16 years (Evans and Gray, 1991) and their growth occurs daily, seasonally and annually; the growth rate is influenced by several environmental and ontogenic factors and can vary significantly (Blackmore, 1969; Ekaratne and Crisp, 1982; Jenkins and Hartnoll, 2001; Fenger et al., 2007). Moreover, the relative percentages of calcite and aragonite in *Patella*, as well as the width of the *m* + 1 aragonitic layer relative to the calcitic *m* + 2 and *m* + 3 layers, have been found to be strongly correlated to relative changes in sea-surface temperature (Cohen and Branch, 1992; Bailey and Craighead, 2003): the higher the aragonite/calcite ratio, the higher the sea-surface temperature.

In our study the two modern shells formed in the cold North Sea waters, as opposed to the two archaeological shells from Gibraltar, which were most likely sourced locally by the Middle Palaeolithic people inhabiting Vanguard Cave. For the modern shells, sea-surface temperatures data are available in a 1° × 1° grid through the NOAA Optimum Interpolation (OI) sea-surface temperature (SST) dataset (NOAA CIRES ESRL/PSD Climate Diagnostics Branch, Boulder, Colorado, 2006; http://www.cdc.noaa.gov). Shell SM3 was collected alive in 2001, so its mean SST, calculated on a grid around the sampling location (54.5–55.5°N, 1–2°W, see Fenger et al., 2007), varied seasonally between ∼18 °C (Jun–Aug 2000) and ∼11 °C (Nov 2000–Feb 2001). SST values for shell C2 were calculated on a 54.5–55.5°N, 0–1°W grid for the summer (Jun–Aug 2006, mean SST ∼19 °C) and winter (Nov 2006–Feb 2007, mean SST ∼11 °C) months of the year prior to collection.

Monthly average SSTs from 1990 to 2008 for Gibraltar ranging between 15.5 and 23.3 °C are reported in Ferguson et al. (2011) and were obtained through the same NOAA dataset. However, these estimates may have little bearing for archaeological shells which were collected during the Middle Palaeolithic, when the SSTs were different due to global climate changes. Between the Last Interglacial (∼125 ka BP) and 40 ka BP the global oxygen isotope record records great climatic variability during MIS 4 and MIS 3. Ferguson et al. (2011) analysed *Patella* shells from archaeological deposits in the Gibraltar caves for Mg/Ca and oxygen isotope ratios and estimated past seasonality in SSTs. They detected a clear trend of cooling between 40 and 19 ka BP, with increased seasonal ranges than have been modelled to date. Shells dated to ∼29 ka BP, hence collected during a glacial stage, yielded estimated SSTs between ∼10 and ∼20 °C (Ferguson et al., 2011). Although the true age of the Vanguard Cave specimens is unknown, they are likely to have been collected prior to 40 ka, during MIS 3. If this is the case, then the relative thickness of the aragonitic layers within these specimens could be explained in terms of relatively warmer SSTs in Gibraltar than in the UK during shell calcification (e.g. Cohen and Branch, 1992). However, other factors may affect this result, including geographical variations (morphological/genetic) in populations of the same species, as well as small-scale environmental and large-scale climatic changes.

In this study we did not investigate further the relationship between thickness of the aragonitic layers in *P. vulgata* and SSTs, but we suggest that two-dimensional XRD mapping would offer an alternative to the traditional techniques for quantifying the aragonite:calcite ratio. This method does not rely on the use of staining solutions, which can introduce a high level of uncertainty in the measurement because of the “fuzziness” of the boundaries between calcitic and aragonitic layers (see Bailey and Craighead, 2003), and yields high-resolution data which are comparable across specimens. Therefore, the accurate quantification of the aragonite:calcite ratio in archaeological *Patella* by XRD mapping could represent an independent source of information which could be used in conjunction with elemental isotope analyses for estimating SSTs in the past.

#### 3.1.2 Sampling strategy

Past AAR studies on *Patella* shells have targeted either the rim (e.g. Davies, 1983) or the apex (e.g. Ortiz et al., 2009) of the shell; the rims have also been used for stable isotope analyses and SST reconstructions in the studies of Fenger et al. (2007) and Ferguson et al. (2011). We devised our sampling strategy on the basis of a number of observations. In general, the distribution of the microstructural layers around the apex area of *P. vulgata* appears to be variable. Moreover, erosion of the shell surface during the animal growth and as a result of post-depositional and burial processes can cause layers to become exposed in concentric bands: the outermost layer can be visible only as a thin ring at the very edge of the specimen, while at the apex exposure of the innermost layer can occur (MacClintock, 1967). The total loss of the apex in archaeological *Patella* shells is also frequently observed, resulting from preferential fracture at the interface between the apex and the rest of the shell. This fracture line corresponds with the variation in the growth angle occurring during the life of the organism as a response to different habitats (Craighead, 1995, pp. 87, 114).

Therefore, for archaeological and geological shells (especially where age and palaeoenvironmental conditions of growth are unknown) we suggest that the shell rim should be targeted for AAR analysis due to its simpler microstructure: only the calcitic layers *m* + 2 and *m* + 3 should be present.

However, the thin aragonitic layer present on the external surface and detected by XRD mapping in this study could potentially hamper the ability of the entire rim to act as a stable mineral fraction to protect the intra-crystalline proteins. Therefore, we devised a sampling procedure which involves subsampling of the shell rim by snapping off a fragment with a surface area of few mm^2^, followed by removal of the thin outer layer of aragonite (Fig. 2). This was done by lightly drilling the surface, using a clean diamond tip, until the calcitic layer below (white in colour) was fully exposed. This layer was then targeted for IcPD AAR. This sampling procedure was adopted for the AAR analyses of *Patella* shells from archaeological and geological sites in both the UK and Southern Europe (Demarchi, 2009; Demarchi et al., 2011).

**Fig. 4:**
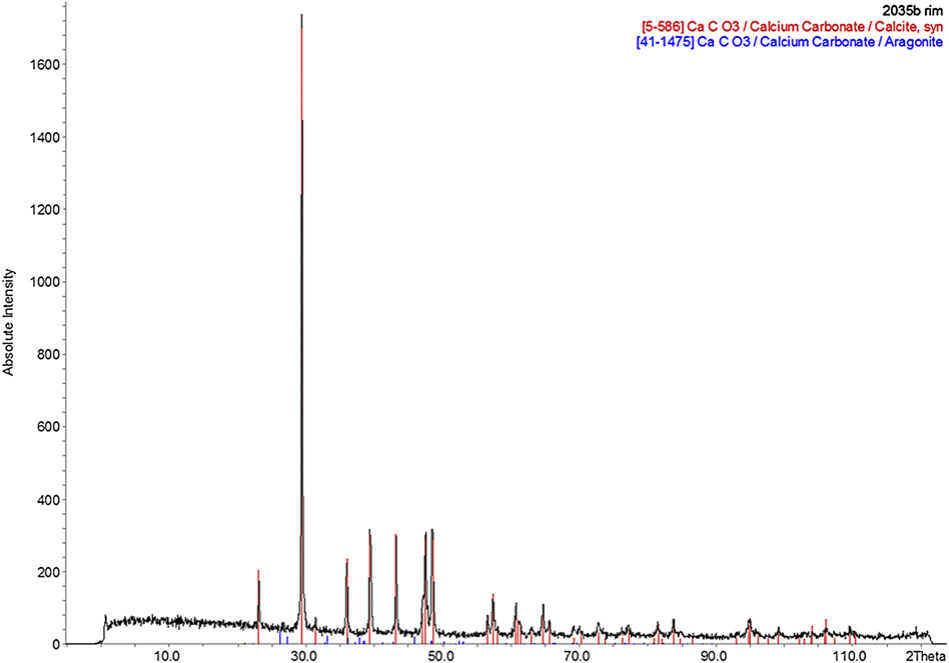
Powder XRD spectrum obtained on a subsample of the rim of specimen LOT 2035 (provenance: Vanguard cave, Gibraltar), after removal of the outer aragonitic layer. Note the absence of any intense peak attributable to aragonite.

We tested the successful removal of all aragonite from the rim subsamples by performing additional XRD analyses on powdered subsamples removed from three individual *Patella* shells from archaeological deposits in Gibraltar. A subsample from specimen LOT 2035, discussed above, was included in these analyses and confirmed the presence of calcite alone in the powder XRD spectrum (Fig. 4). This confirmed that the sampling strategy chosen in this study is able to isolate a portion of the *Patella* shell which is composed of only calcite and therefore with the potential to act as a good repository for the intra-crystalline proteins over geological timescales.

### 3.2 Bleaching tests

The intra-crystalline proteins are defined operationally as the fraction of proteins which is not removed after prolonged strong oxidation, as opposed to inter-crystalline (matrix) proteins (Sykes et al., 1995; Penkman et al., 2008). A simplified theory for explaining their resistance to the oxidation agents involves the entrapment of the proteins within the mineral crystals (see Towe, 1980; Sykes et al., 1995; Penkman et al., 2008). Whether these proteins can truly be encapsulated within the mineral crystals is at present unclear, but new crystallisation theories (e.g. Cölfen, 2008) may offer the opportunity to understand their spatial location.

We performed bleaching experiments on modern bulk powder of *P. vulgata* (fine, medium and coarse size fractions, as detailed in Section 2.3.1) in order to establish:

1) the effectiveness of NaOCl (bleach) as an oxidising agent in isolating a fraction of intra-crystalline proteins from different size fractions of the powdered shell;
2) the stability of the intra-crystalline fraction after prolonged exposure to NaOCl;
3) the optimum bleaching time, i.e. the shortest exposure to NaOCl which is effective in isolating the intra-crystalline proteins.

#### 3.2.1 Effectiveness of preparative hydrolysis on *P. vulgata*

Testing the effectiveness of bleaching in removing matrix protein requires an accurate quantification of the amino acid concentrations within both unbleached and bleached shells. In this study we routinely use a 24-h acid hydrolysis step, performed at 110 °C and by exposing the shell powders to 7 M HCl. This pre-treatment should be able to release all amino acids from the peptide chain and to allow the accurate estimation of the concentration of the total hydrolysable amino acids (THAAp fraction, here referred to as THAA).

**Fig. 5:**
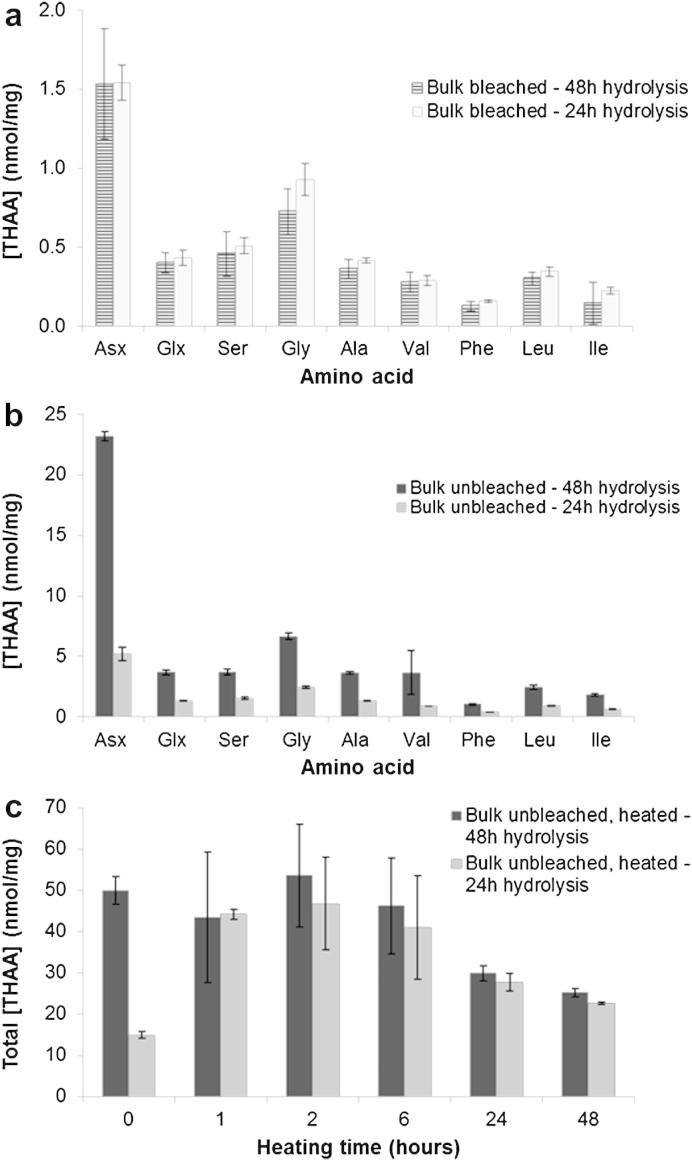
(a) [THAA] values recovered from bleached *P. vulgata* bulk powders (medium-to-fine fraction) after a 24-h and a 48-h acid hydrolysis step; (b) [THAA] values recovered from unbleached *P. vulgata* bulk powders (medium-to-fine fraction) after a 24-h and a 48-h acid hydrolysis step; (c) Total [THAA] values detected in unbleached *P. vulgata* bulk powders (medium-to-fine fraction) heated at 140 °C for different times and hydrolysed for either 24 or 48 h; note that the differences in concentration become negligible after 1 h heating at 140 °C. Total [THAA] values in c are calculated as the sum of [THAA] of Asx, Glx, Ser, Gly, Ala, Val, Phe, Leu, Ile. Error bars represent one standard deviation around the mean for the laboratory replicates.

In order to test this, we attempted longer hydrolysis treatments on bleached and unbleached *P. vulgata* powders (medium-to-fine size only). A 48-h acid hydrolysis step did not induce any significant changes to the THAA composition of bleached powders compared to a 24-h hydrolysis step (Fig. 5a); therefore a 24-h hydrolysis treatment is sufficient for releasing protein-bound amino acids in bleached shells. On the contrary, a 48-h step produced a significant increase in [THAA] in unbleached powders for most of the amino acids considered here (Fig. 5b). To our knowledge, such a striking effect of preparative hydrolysis on the yield of THAA has never been reported for molluscan shell proteins. However, the difference between a 24-h and a 48-h hydrolysis step for the unbleached powders is significant only for unheated samples. Bulk powders used for leaching experiments (Section 3.3) and heated at 140 °C for various times (see Table 1) were hydrolysed for 24 h and 48 h and the [THAA] values compared (Fig. 5c); the yield of THAA was found to be similar between the two sets of samples when exposed to high temperatures for at least 1 h (Fig. 5c). This suggests that diagenesis (either temperature-induced or natural) affects the solubility of the matrix protein complex in unbleached *P. vulgata* and that a 24-h acid hydrolysis step is appropriate for the analysis of fossil shells as well as shells heated at high temperatures, whilst a 48-h step is necessary for unbleached unheated modern *P. vulgata* shells. Therefore, the discussion of the results obtained on the bleaching and leaching experiments (Sections 3.2.2 and 3.3) is based on the [THAA] values obtained with the 48-h hydrolysis step only for unbleached, unheated shell powders. All other [THAA] values reported here (bleached powders and unbleached heated powders) were obtained by using a 24-h hydrolysis treatment.

#### 3.2.2 Effectiveness of bleaching

**Fig. 6:**
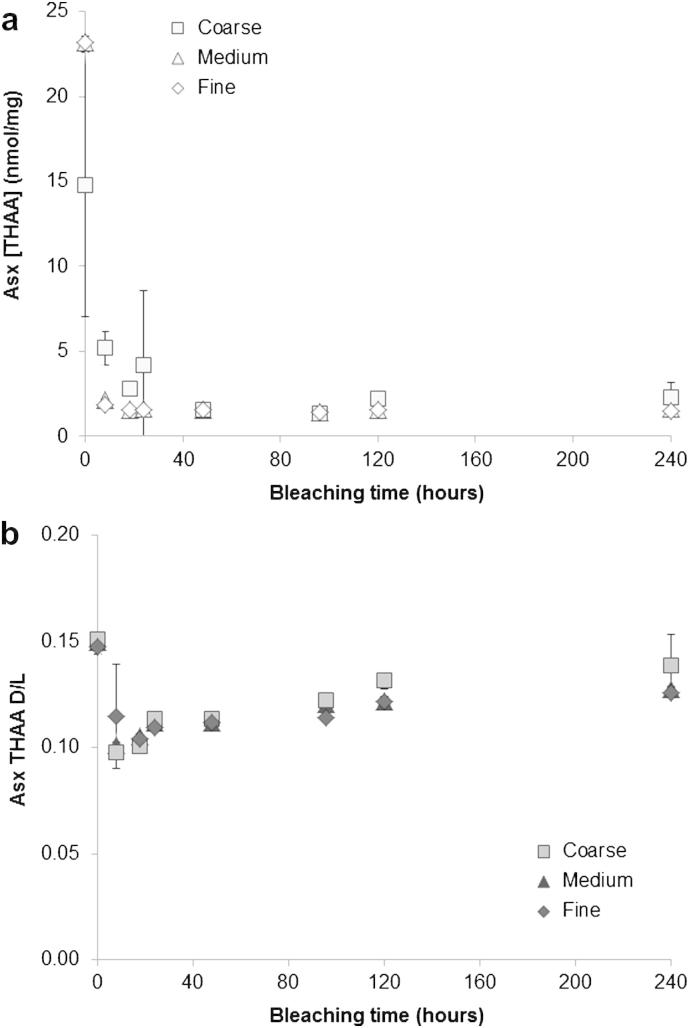
Effect of bleaching time on THAA detected in the bulk powders of *Patella vulgata*; error bars represent one standard deviation around the mean, calculated on three replicates for the same time point. (a) Decrease of Asx [THAA] with increasing bleaching time; note the higher variability displayed by the coarse fraction compared to the medium and fine fractions. The unbleached [THAA] values for the medium and fine powders were obtained by using a 48-h acid hydrolysis step. (b) Asx THAA D/L values against bleaching times. The slight increase in the D/L values after 8 h bleaching indicates an effect of bleaching upon the racemisation reaction; however, this effect is small and it can be observed for Asx and Ser only. The unbleached D/L values were obtained by using a 24-h acid hydrolysis step.

Data from the bleaching experiments show that just a few hours of exposure to NaOCl causes the concentration of all the amino acids considered to fall (THAA Asx data shown in Fig. 6a). A steady pattern of decrease in amino acid concentration with increasing bleaching time was observed for both the fine and the medium fractions of the powdered shell, whilst the values recovered from the coarse fraction are at slightly higher concentrations and are generally more variable (Fig. 6a). This could be due to incomplete bleaching, caused by the difficulty of the oxidising agent to penetrate within the shell ultrastructure (Penkman et al., 2008). Therefore, in the case of *Patella*, it is important that the particle size is taken into account for the successful isolation of the intra-crystalline proteins. Only the medium and fine fractions were targeted for leaching and kinetic experiments (Section 3.3 and Demarchi et al., 2013) and we recommend the selection of this particle size (maximum 500 μm) for further studies on the intra-crystalline proteins in *Patella*.

After 48 h of exposure to the bleaching agent, the THAA concentrations plateaued (Fig. 6a). More prolonged oxidation did not cause a further reduction in THAA concentration. It is hypothesised that the inter-crystalline matrix is oxidised because it is more easily accessible to the bleaching agent, and therefore that the retained amino acids represent the intra-crystalline proteins. Consequently, a 48-h exposure to NaOCl was chosen as the optimal bleaching time for *P. vulgata*.

Contrary to that observed in ostracodes, we did not detect an increase in [THAA] values (see Bright and Kaufman, 2011) with increasing bleaching times. This suggests that all the soluble inter-crystalline proteins within *P. vulgata* are removed by the oxidation treatment; but this does not exclude that a residual fraction of highly insoluble proteins, immune to chemical removal, remains in the system.

Reaction with a strong oxidation agent could also induce a certain amount of racemisation, so it is necessary to determine whether the DL ratios increase with the progression of the treatment. A decrease in the racemisation values within the THAA fraction was observed between the first two bleaching steps (0–8 h) for all amino acids except Val; after 8 h of bleaching, D/L values for Asx and Ser display a slight increase (Asx data shown in Fig. 6b). Generally, D/L values detected from the coarse powders showed higher variability than for the medium and fine powders (Fig. 6b). However, the variation in D/L values upon bleaching is very small and it can be concluded that the pre-treatment with NaOCl did not significantly affect the extent of racemisation and therefore that the intra-crystalline fraction is effectively stable.

#### 3.2.3 Inter- vs intra-crystalline proteins

**Fig. 7:**
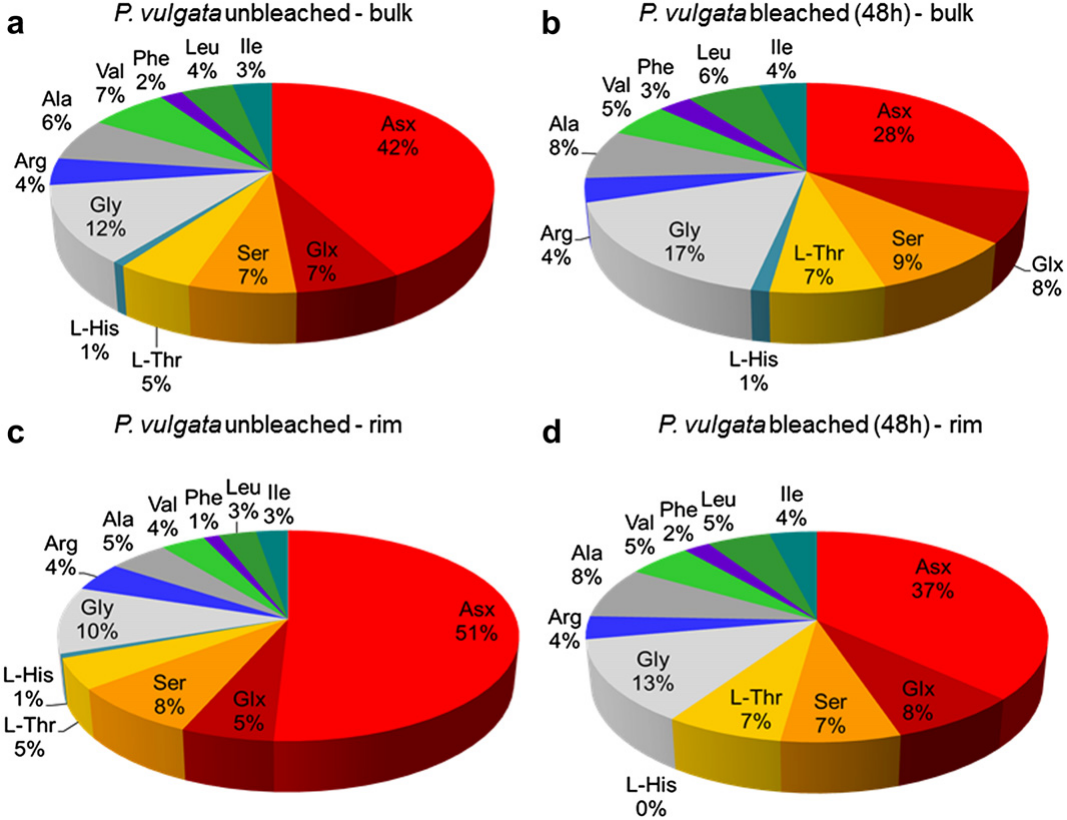
THAA composition for different fractions of *P. vulgata* shells: (a) unbleached bulk powders, medium-to-fine fraction only (values obtained with a 48-h hydrolysis step); (b) bleached bulk powders, medium-to-fine fraction; (c) unbleached rim powders, medium-to-fine fraction only; (d) bleached rim powders, medium-to-fine fraction only.

After 48 h of bleaching only ∼10% of the total original amino acids are retained within the intra-crystalline fraction isolated from the bulk shell (Table 2). A considerable change in amino acid composition is also observed, as Asx represents more than 40% of the total [THAA] in unbleached *P. vulgata* (values obtained after a 48-h hydrolysis step, as described in Section 3.2.1; Fig. 7a) but only 28% in powders which have been bleached for 48 h (Fig. 7b). This is in contrast to that observed for other mollusc shells, where the relative percentage of Asx tends to increase after bleaching (e.g. Penkman et al., 2008). Moreover, the protein composition in *Patella* is different to published intra-crystalline data from gastropods and bivalves (Penkman et al., 2008), confirming that isolation of the intra-crystalline protein does not circumvent the “species effect” in molluscs. However, the intra-crystalline fraction within modern *P. vulgata* is still dominated by acidic proteins, as Asx and Glx together make up ∼35% of the total intra-crystalline amino acids, followed in importance by Gly and Ser (Fig. 7a and b).

#### 3.2.4 Bulk shell vs rim

As proteins regulate biomineralisation, the amino acid compositions are likely to differ between the bulk shell and the rim, due to their different microstructures and mineralogies (see Section 3.1.1). Therefore we characterised the amino acid composition of both the inter-crystalline and the intra-crystalline (isolated by a 48-h bleaching step) fractions from the calcitic rim of modern *Patella* shells and compared it to the composition of the proteins isolated from the bulk shell. For the “rim only” samples, the medium-to-fine fraction powder only was selected, as this yielded the best results for the bleaching experiments performed on the bulk shell (see Section 3.2.2). The matrix (inter-crystalline) proteins from the unbleached rim were effectively hydrolysed by a 24-h acid hydrolysis step; a 48-h step did not yield significantly different [THAA] values (see Supplementary Information).

**Fig. 8:**
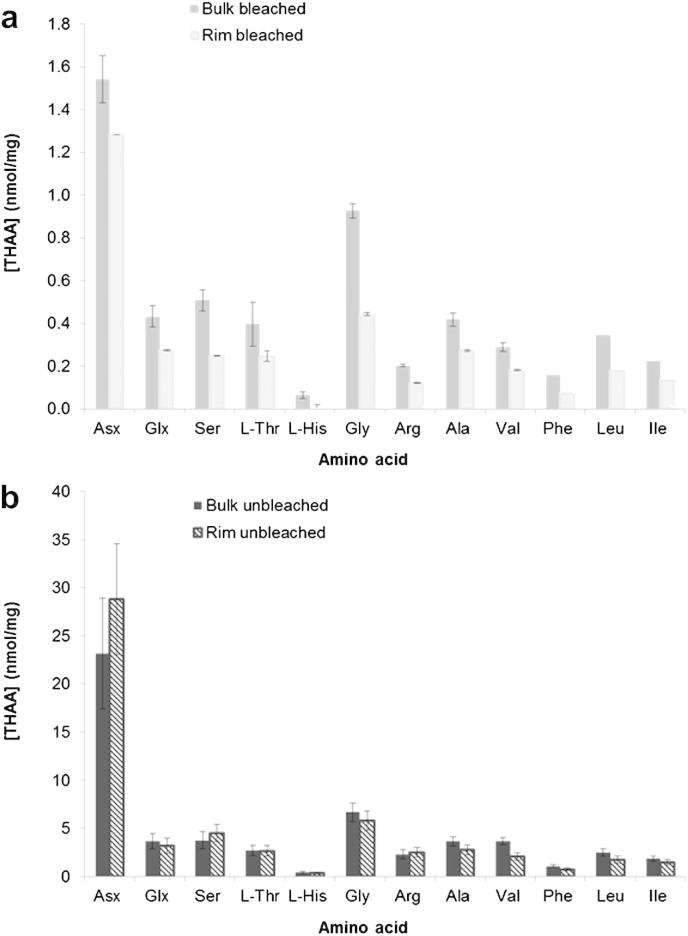
Comparison between the THAA concentrations of the (a) intra-crystalline (bleached) and (b) matrix (inter- and intra-crystalline, unbleached) amino acids isolated from the “bulk” shell sample and the rim only (medium-to-fine powder fraction). Error bars represent one standard deviation around the mean value for each amino acid.

The amino acid concentrations are slightly higher in the *unbleached* rim (∼57 nmol/mg) than in the unbleached bulk (∼50 nmol/mg); conversely, *bleached* [THAA] values are lower in the shell rim than in the bulk shell fraction (Fig. 8). Bleached powders from the bulk shell yielded a maximum value for the total concentration of ∼4.3 nmol of amino acids per mg of powder (Table 3). This indicates that the intra-crystalline fraction represents ∼0.55% by weight of the bleached bulk powder, assuming an average molecular weight of 125 g/mol for each amino acid and limiting the calculation to the 9 amino acids considered here. The concentration recovered from the rim only is ∼2.8 nmol/mg (Table 3), therefore representing ∼0.35% by weight of the bleached powder. The THAA D/L values detected for Asx, Glx, Ser, Ala and Val are slightly higher in the bleached rim than in the bleached bulk shell (see Supplementary Information). This is likely to be related to differences in relative composition between the two and may indicate that the intra-crystalline proteins retained in the rim are more prone to breakdown than the ones retained in the bulk shell.

The relative percentage of Asx is higher in the rim than in the bulk, for both unbleached and bleached powders (Fig. 7). Acidic proteins are usually found in association with calcite biominerals, although the reason for this selection remains unknown (Marin et al., 2007). The intra-crystalline proteins within *Patella* rims (Fig. 7d) show similar composition to that of a group of proteins (Asp-rich, UNIPROT entry: Q5Y821_ATRRI) isolated from the prismatic calcitic shell matrix of the bivalve *Atrina rigida* (Gotliv et al., 2005); however, the latter display higher relative percentages of Ala compared to Gly than *Patella* (both rim and bulk). High percentages of Asx in the rim proteins might enhance breakdown due to the facile in-chain racemisation of Asx (Geiger and Clarke, 1987) and therefore account for the higher D/L values recovered from the rim samples.

#### 3.2.5 Conclusions on bleaching experiments

Bleaching experiments performed on *P. vulgata* showed that both bulk shell powders and the calcitic rim retain a fraction of proteins which can be effectively isolated by a 48-h bleaching step, whilst inducing a negligible amount of racemisation. The intra-crystalline protein concentrations are relatively high (3–5 nmol/mg), allowing precise quantification via RP-HPLC without the interference of the background noise (generally ∼2–10 pmol/mg).

The composition of both inter- and intra-crystalline proteins within *Patella* is dominated by acidic amino acids; however, the relative percentages of Asx vary between the whole-shell and the intra-crystalline fraction. Intra-crystalline proteins isolated from the rim only and the bulk shells also display different compositions. As a consequence, although different sampling strategies can be used for different biominerals, it is important that the sampling location is consistent for all specimens considered for IcPD studies, in order to be able to compare results directly.

### 3.3 Leaching tests

Leaching tests have proven a simple and reliable way to assess the suitability of the intra-crystalline proteins isolated by bleaching for closed-system AAR geochronology. The stability of intra-crystalline protein during diagenesis is tested by exposing the bleached powder in an hydrous environment to high temperatures; if leaching (or diffusive loss) does occur upon heating, the amino acids will be detectable in the supernatant water and this will indicate an open-system behaviour (e.g. Penkman et al., 2008; Demarchi et al., 2011). For AAR geochronology ideally the system should remain closed from the death of the organism until the recovery of the fossil from its burial environment and subsequent analysis; significant leaching therefore indicates that the extent of diagenesis detected within a biomineral behaving as an open system does not necessarily represent the extent of degradation of the indigenous protein fraction.

Here we discuss the different behaviours of unbleached and bleached shells, allowing the comparison of the conventional AAR method and the IcPD approach. In addition we compare the behaviour of the intra-crystalline proteins isolated from the shell rim only and those isolated from the bulk *P. vulgata* powders to assess the potential of both for preserving the intra-crystalline proteins.

#### 3.3.1 Loss of open-system amino acids: quantification

Amino acids lost by leaching out of the system upon isothermal heating can be quantified on the basis of the concentrations recovered from the powders (THAAp and FAAp fractions; both unbleached and bleached) and the waters in which the powders have been heated (THAAw and FAAw fractions) for various times and at different temperatures (Section 2.3.2; Table 1; Fig. 1)

**Fig. 9:**
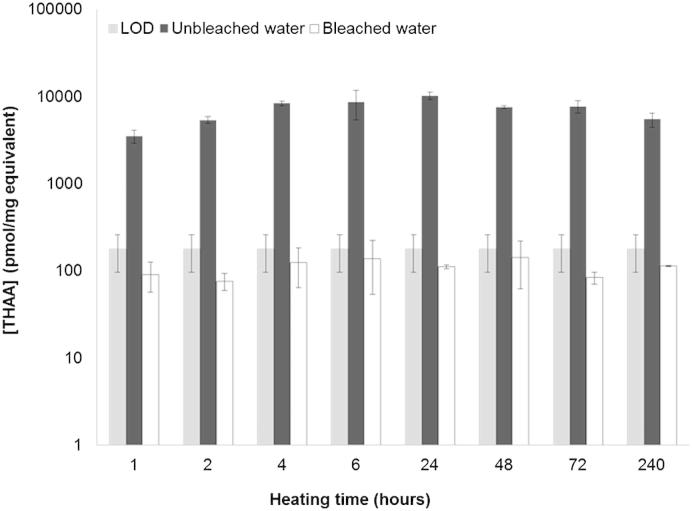
Total concentration of amino acids recovered from the water from bleached and unbleached bulk *Patella* powders upon heating at 140 °C. Each bar represents the sum of the THAA concentration of Asx, Glx, Ser, Gly, Ala, Val, Phe, Leu and Ile. Error bars represent one standard deviation around the mean, calculated on three replicates for the same time point. The limit of detection was calculated on the basis of the concentrations recovered from procedural blanks analysed together with the samples: LOD = *X*_blank_ + *k* ^∗^ *σ*_blank_; where *X*_blank_ is the mean of the blank measures, *σ*_blank_ is the standard deviation of the blank measures, and *k* = 3.

For bleached bulk *Patella* shells the concentration of amino acids in the water was similar to background levels; this observation was verified in THAAw and FAAw samples heated for various times at high temperature. Here we define as background level the limit of detection (LOD), calculated on the basis of the concentrations recovered from procedural blanks analysed together with the samples and shown in Fig. 9. In contrast, the concentration of amino acids in the water for unbleached shells was an order of magnitude higher (nanomoles/mg equiv as opposed to picomoles/mg equiv) throughout the duration of the experiment at 140 °C (Demarchi et al., 2011 and Fig. 9).

**Fig. 10:**
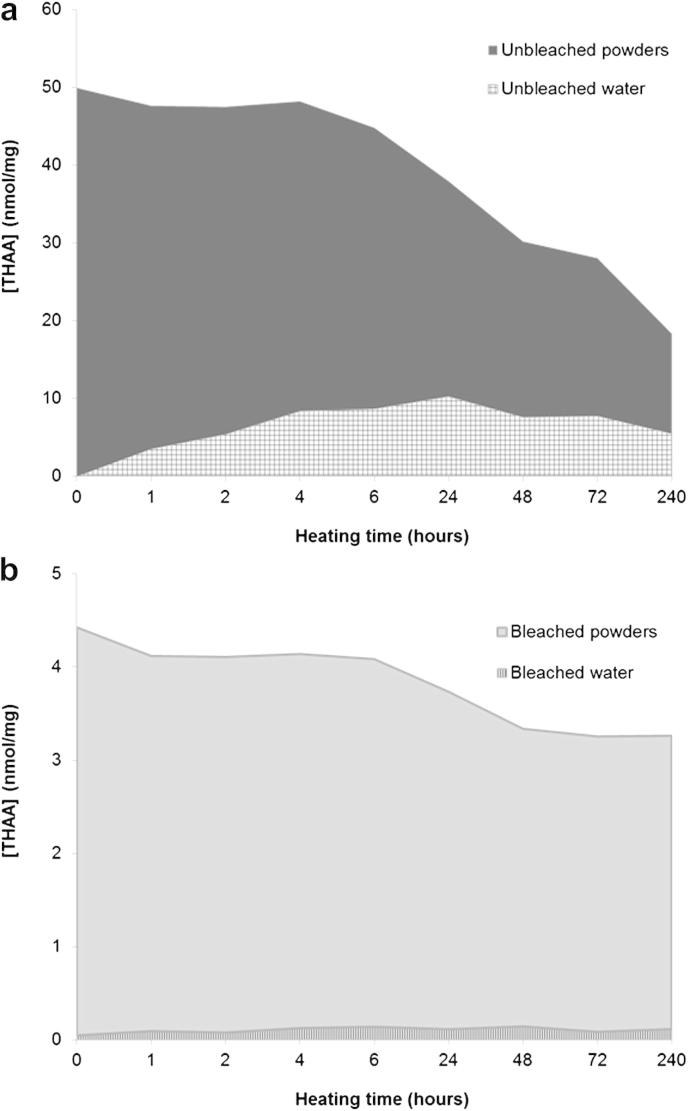
(a) Total concentrations of amino acids in unbleached *Patella* shell powders (bulk) and supernatant water. (b) Total concentrations of amino acids in bleached *Patella* shell powders (bulk) and supernatant water. Areas represent the sum of the [THAA] of the following amino acids: Asx, Glx, Ser, Gly, Ala, Val, Phe, Leu, Ile (different scales on the y-axis for a and b). Note that the minimum concentration in unbleached powders (reached after 240 h heating) is approximately three times higher than the concentration within the intra-crystalline fraction at any given time point.

Bleaching experiments showed that unbleached *Patella* contains both matrix (inter-) and intra-crystalline proteins; leaching experiments show that upon isothermal heating some inter-crystalline proteins are lost from the unbleached powder and are released in the water: the [THAAp] values decrease with increasing heating time, as the amino acids are leached out of the shell or lost due to decomposition (the network of processes leading to the chemical degradation of the molecular structure of the amino acids). An increase in [THAAw] can be observed, confirming that leaching is taking place in the system (Fig. 10a). If leaching continues, the total concentration of amino acids in the unbleached powders would eventually reach the concentration levels detected in bleached powders, i.e. prolonged leaching would isolate the intra-crystalline fraction. This was not observed under the experimental conditions used here: after 240 h heating at 140 °C the [THAA] in unbleached powders is still three times higher than in the intra-crystalline fraction (Fig. 10a and b). However, it is likely that this process would occur over geological timescales (see also Miller and Hare, 1980; Collins and Riley, 2000; Bright and Kaufman, 2011).

Contrary to the unbleached powders, the intra-crystalline fraction remains generally closed over time during isothermal heating: a decrease in bleached [THAAp] can be observed, but it does not correspond to a comparable increase in [THAAw] (Fig. 10b). Therefore, the main loss of amino acids within the intra-crystalline fraction is due to decomposition.

**Fig. 11:**
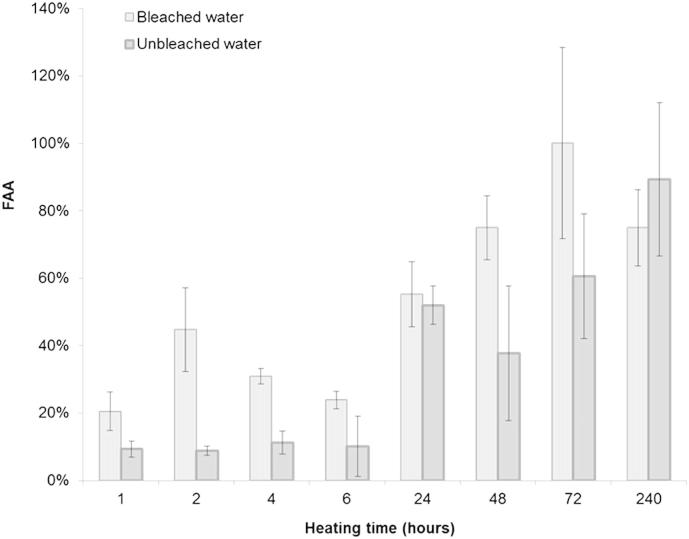
%FAA recovered from the water used in the heating experiment for both unbleached (dark grey area) and bleached (light grey area) *Patella* bulk shell powders. %FAA values were calculated respect to the THAA concentrations recovered from the water samples. Values represent the sum of the concentrations of the following amino acids: Asx, Glx, Ser, Gly, Ala, Val, Phe, Leu, Ile.

For unbleached shells, only a small percentage of amino acids recovered in the water are in the free state within the first 24 h of heating (Fig. 11), indicating that amino acids are lost while still peptide-bound and then undergo hydrolysis in the aqueous environment when exposed to high temperatures for longer times. The same pattern can be observed for the bleached shells; however, the concentration values of the amino aids lost in the water from bleached shells are too low for drawing any conclusion as to whether peptide-bound amino acids are truly lost in the water.

The loss of amino acids after 24 h and 48 h heating at 140 °C from both bleached and unbleached powders is reported in Table 3. After 24 h, more than 20% of the initial total content of whole-shell amino acids is lost in the water, while, on average, only 1% of the proteins within the intra-crystalline fraction are lost by leaching (after blanks subtraction, see Table 3). In general, these results are in concordance with those obtained on terrestrial gastropods, where only 0.7% of the initial protein content was lost by leaching under the same experimental conditions (Penkman et al., 2008). After 48 h at 140 °C, the percentage of amino acids lost into the water is ∼3% for the bleached bulk shell; on the other hand, the contribution of leaching is slightly higher for the rim samples (∼4%; after blanks subtraction, see Table 3)

For both unbleached and bleached shells, the contribution of leaching is consistently lower than the overall loss of amino acids observed in the powders after heating; the difference between the overall loss and the loss due to leaching is attributed to the decomposition of the amino acids in the system. It is interesting to note that after 48 h heating at 140 °C the bleached rim samples had lost only 8% of the original total amino acids by decomposition, while the bleached bulk shell had lost 24%.

However, these estimates rely heavily on the precision of the [THAAw] values; if a one standard deviation interval is considered for THAAw values recovered from samples heated 48 h, the contribution of leaching varies between 0 and 4% for bulk bleached shells and between 2 and 6% for the bleached rim only, and the estimates of the loss of amino acids by decomposition will vary accordingly. The uncertainty is generally higher for bleached samples because the [THAAw] values are close to the limit of detection (LOD). Moreover, decomposition also affects amino acids in the water; therefore the [THAAw] detected at a given time point represent a minimum estimate of the loss of amino acids by leaching.

#### 3.3.2 Behaviour of matrix and closed-system proteins

The suitability of the intra-crystalline proteins isolated from any biomineral for IcPD geochronology can be tested further by investigating their diagenetic behaviour. Here we briefly examine the extent of racemisation and decomposition of multiple amino acids extracted from both bleached and unbleached *P. vulgata*, heated at 140 °C. The diagenesis patterns displayed by the intra-crystalline fraction within *Patella* are described in detail in another study (Demarchi et al., 2013).

**Fig. 12:**
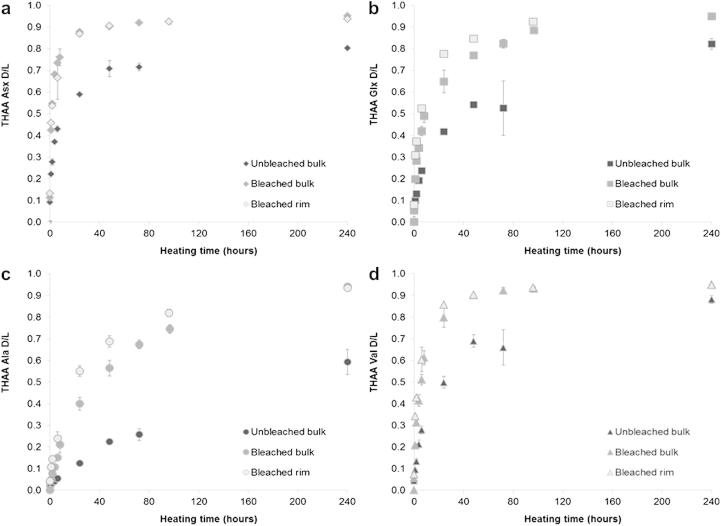
Extent of racemisation detected in unbleached (bulk) *Patella* shells compared with bleached samples (both bulk and rim) upon heating at 140 °C: (a) Asx, (b) Glx, (c) Ala and (d) Val.

**Fig. 13:**
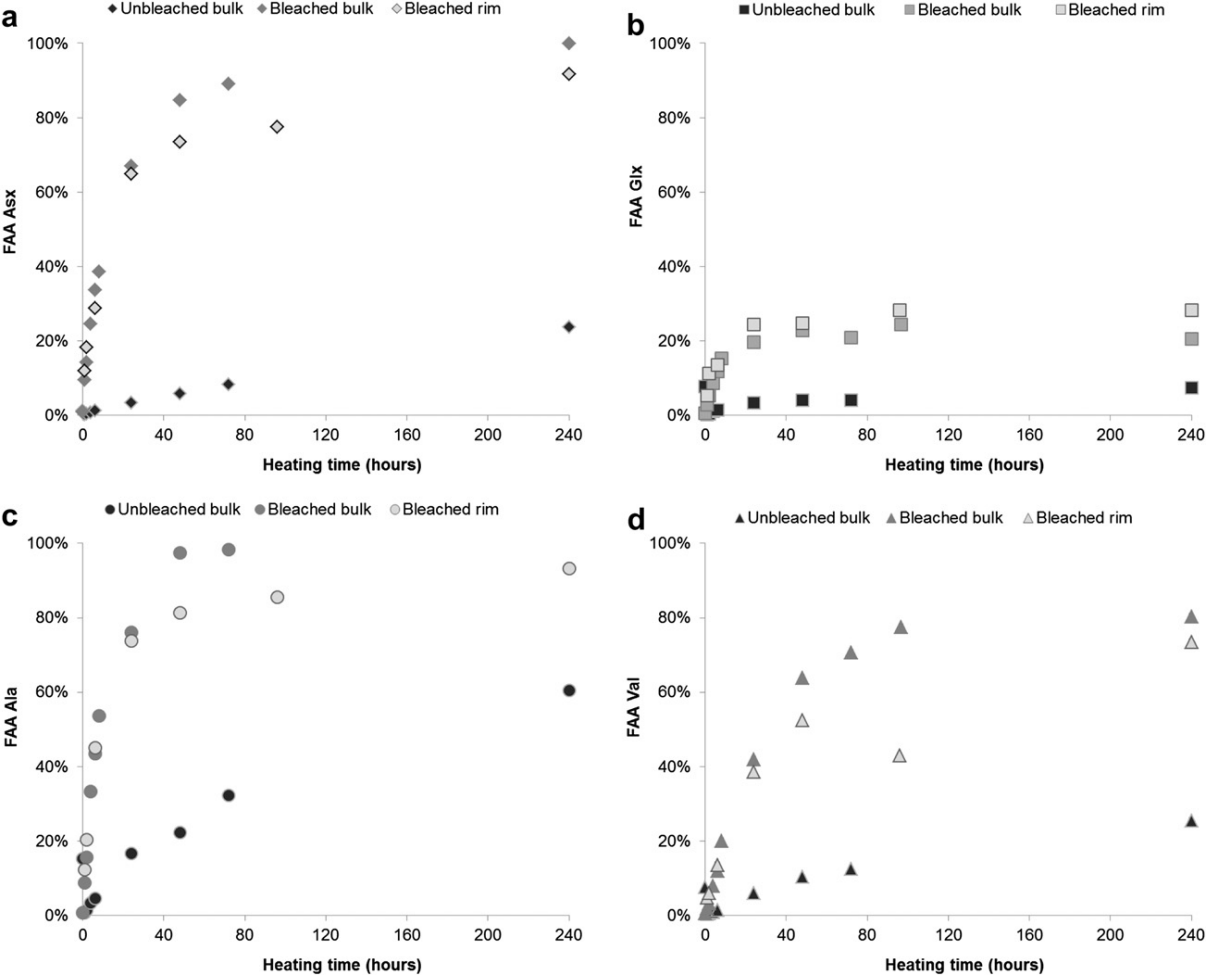
Extent of release of free amino acids detected in unbleached (bulk) *Patella* shells compared with bleached samples (both bulk and rim) upon heating at 140 °C: (a) Asx, (b) Glx, (c) Ala and (d) Val.

The extent of racemisation (Fig. 12) and the %FAA (Fig. 13) were found to be consistently higher in bleached than in unbleached shells. After 48 h heating at 140 °C, unbleached Val only reached a THAA D/L value of 0.24, while bleached shells display THAA D/L values of 0.56 (bulk shell) or 0.69 (edge only) (Fig. 12); %FAA Val at the same time point is ∼65% for bleached shells but only ∼10% for unbleached shells (Fig. 13). Therefore the higher extent of racemisation detected in bleached shells may be due to the higher proportion of more racemised FAA retained within the intra-crystalline fraction (see the model by Penkman et al., 2008).

When the extent of intra-crystalline racemisation for the two subsampling locations is compared, the D/L values measured on the rim appear to be slightly higher than the ones measured on the bulk shell. This is more evident for the slow-racemising amino acids, such as Glx, Val and Ile. As the protein composition is different between the two subsampling locations (Fig. 7), the degradation pathways may vary, particularly with regards to the relative rates of hydrolysis of the peptide backbone and the likelihood of the exposure of slow-racemising amino acids at terminal positions, where racemisation is accelerated; this would therefore result in higher extent of racemisation.

#### 3.3.3 Closed-system behaviour

The amino acid fraction within unbleached shells display marked open-system behaviour, as ∼20% of the total amino acid concentration is rapidly lost in the water upon heating; on the contrary, the loss of amino acids from the intra-crystalline fraction was limited and the [THAAw] values detected in the water were comparable to the limit of detection. The bleached bulk shell powder appear to be slightly less affected by leaching than the bleached rim (3% THAA lost from the bulk vs 4% lost from the rim after 48 h heating); however, due to the uncertainty affecting the concentration values similar to the LOD, this difference is not significant and the two systems can be considered to display a similar behaviour with regard to leaching.

In general, the intra-crystalline protein within *P. vulgata* show some evidence of slight leaching, therefore indicating that the system is not completely closed and there may be a small exchange of amino acids between the intra-crystalline fraction and the surrounding environment can occur, similar to that observed for terrestrial gastropods (Penkman et al., 2008). However, the vast majority of the protein isolated after bleaching does not leach and the higher extent of amino acid racemisation observed in bleached shells shows that the intra-crystalline fraction is able to retain free amino acids in the system. Therefore, these proteins better approximate a closed system than the whole-shell fraction, and can be considered to be a suitable substrate for IcPD studies.

## 4 Conclusions

This paper has evaluated the potential of marine gastropods *P. vulgata* for IcPD geochronology. XRD mapping has been applied to examine the mineralogy of the shells and this has identified the calcitic rim of the shell as the most appropriate subsampling location for this shell taxon.

The intra-crystalline proteins from both a bulk shell sample and the rim only were isolated by bleaching and tested for closed-system behaviour during diagenesis by isothermal heating at high temperature, whilst leaching experiments were performed in order to detect loss of proteins from the system. Results from both tests show that the intra-crystalline proteins within modern *P. vulgata* show limited leaching and therefore approximate a closed system with regard to protein diagenesis. In contrast, unbleached samples were very prone to leaching, exhibiting typical open-system behaviour. The racemisation patterns vary between unbleached and bleached shells, but also between the intra-crystalline proteins isolated from two subsampling locations. Therefore, whilst *Patella* appears to be a well-suited taxon for IcPD geochronology, we recommend that the intra-crystalline fraction is isolated from a consistent location within the calcitic portion of the shell rim.

## Figures and Tables

**Table 1:** Details of the method used for heating experiments: temperature (°C), bleached & unbleached, bulk shell & rim, heating times used for each experiment (h).

Powder fraction	Temperature	Heating time (h)										
Bleached, bulk	140 °C	0	1	2	4	6	8	24	48	72	97	240
Bleached, rim only	140 °C	0	1	2		6		24	48		97	240
Unbleached, bulk	140 °C	0	1	2	4	6		24	48	72		240

**Table 2:** Percentage of amino acids retained within the intra-crystalline fraction in modern *P. vulgata* after 48 h bleaching (“bulk” and “rim only” shell samples, medium-to-fine powder fraction only).

[THAA]	Asx	Glx	Ser	Gly	Ala	Val	Phe	Leu	Ile
% Retained in bulk sample	7	12	14	14	11	8	15	14	12
% Retained in rim sample	4	9	6	8	10	9	10	10	9

**Table 3:** Loss of amino acids upon heating from bleached (B) and unbleached (U) *P. vulgata*. [Total] represents the sum of [THAA] Asx, Glx, Ser, Gly, Ala, Val, Phe, Leu and Ile. [THAA] values recovered from the procedural blanks analysed together with the samples were subtracted from all [THAAp] and [THAAw] values included in this calculation.

Heating time/shell fraction	After 24 h @ 140 °C		After 48 h @ 140 °C		
U (bulk)	B (bulk)	U (bulk)	B (bulk)	B (rim)
Initial [Total] THAA in shell, unheated (pmol/mg) (Mean ± *σ*)	49,879 ± 3361	4319 ± 250	49,879 ± 3361	4319 ± 250	2785 ± 22
[Total] THAA in shell, heated (pmol/mg)(Mean ± *σ*)	27,589 ± 1524	3563 ± 826	22,519 ± 304	3135 ± 305	2446 ± 66
[Total] THAAw in water, heated (pmol/mg equiv) (Mean ± *σ*)	11,422 ± 1082	52 ± 6	8482 ± 310	83 ± 80	107 ± 48
Overall loss in shell (%)	45	17	55	27	12
Loss by decomposition (%)	22	16	38	24	8
Loss into water (%)	23	1	17	3	4
